# Genomewide Expression Analysis in Zebrafish *mind bomb* Alleles with Pancreas Defects of Different Severity Identifies Putative Notch Responsive Genes

**DOI:** 10.1371/journal.pone.0001479

**Published:** 2008-01-23

**Authors:** Ashok Hegde, Nick Chuanxin Qiu, Xuehui Qiu, Steven Hao-Kee Ho, Kenny Qi-Ye Tay, Joshy George, Felicia Soo Lee Ng, Kunde Ramamoorthy Govindarajan, Zhiyuan Gong, Sinnakaruppan Mathavan, Yun-Jin Jiang

**Affiliations:** 1 Laboratory of Developmental Signalling and Patterning, Institute of Molecular and Cell Biology, A*STAR (Agency for Science, Technology and Research), Singapore, Singapore; 2 Genome Institute of Singapore, A*STAR (Agency for Science, Technology and Research), Singapore, Singapore; 3 Bioinformatics Institute, A*STAR (Agency for Science, Technology and Research), Singapore, Singapore; 4 Department of Biological Sciences, National University of Singapore, Singapore, Singapore; 5 Department of Biochemistry, National University of Singapore, Singapore, Singapore; 6 School of Biological Sciences, Nanyang Technological University, Singapore, Singapore; University College Dublin, Ireland

## Abstract

**Background:**

Notch signaling is an evolutionarily conserved developmental pathway. Zebrafish *mind bomb* (*mib*) mutants carry mutations on *mib* gene, which encodes a RING E3 ligase required for Notch activation via Delta/Jagged ubiquitylation and internalization.

**Methodology/Principal Findings:**

We examined the *mib* mutants for defects in pancreas development using *in situ* hybridization and GFP expression analysis of pancreas-specific GFP lines, carried out the global gene expression profile analysis of three different *mib* mutant alleles and validated the microarray data using real-time PCR and fluorescent double *in situ* hybridization. Our study showed that the *mib* mutants have diminished exocrine pancreas and this defect was most severe in *mib^ta52b^* followed by *mib^m132^* and then *mib^tfi91^*, which is consistent with the compromised Notch activity found in corresponding *mib* mutant alleles. Global expression profile analysis of *mib* mutants showed that there is a significant difference in gene expression profile of wt and three *mib* mutant alleles. There are 91 differentially expressed genes that are common to all three *mib* alleles. Through detailed analysis of microarray data, we have identified several previously characterized genes and some putative Notch-responsive genes involved in pancreas development. Moreover, results from real-time PCR and fluorescent double *in situ* hybridization were largely consistent with microarray data.

**Conclusions/Significance:**

This study provides, for the first time, a global gene expression profile in *mib* mutants generating useful genomic resources and providing an opportunity to identify the function of novel genes involved in Notch signaling and Notch-regulated developmental processes.

## Introduction

The Notch pathway is an evolutionarily conserved signal transduction cascade that plays essential roles in a variety of developmental processes, such as pattern formation, cell fate determination and organ formation through local cell-cell interactions (reviewed in [1]–[2]). Apart from being important for normal development, Notch signaling is also related to several human congenital diseases, such as T-cell acute lymphoblastic leukemia/lymphoma [3]–[5], Alagille syndrome [6]–[8], a late onset neurological disease (CADASIL) [9], and spondylocostal dysostosis [10].

Notch receptor functions as a membrane-bound transcription factor that turns on specific genes in response to physiological cues that trigger ligand binding. Upon functional binding of DSL (Delta, Serrate/Jagged and Lag-2) transmembrane ligands, the membrane-bound Notch is proteolyzed by TNFα-converting enzyme (TACE), metalloproteases [11] and Presenilin, and the active form–Notch intracellular domain (NICD)–is released [12], [13]. The NICD is then translocated to the nucleus [14]–[17] and binds to the conserved CSL (CBF1/RBPjκ, Su(H) and Lag-1) DNA-binding protein [18], [19], which is converted from a transcriptional repressor to activator. This triggers the expression of downstream target genes such as Hes/Her (hairy/Enhancer of split related) family of bHLH transcription factors, which in turn modulate the expression of downstream genes and themselves [16], [20]–[26].

Several E3 ligases, such as Mind bomb (Mib), Su(dx), Sel-10, Neuralized, and Deltex, have been shown to modulate the Notch signaling through ubiquitin-dependent protein degradation and/or endocytosis [27], [28]. Mib is a ubiquitin ligase that is required cell non-autonomously for Notch signaling and lateral inhibition by controlling Delta protein internalization [27]. Several mutants of zebrafish Notch signaling components, such as *after eight (aei)/deltaD*, *deadly seven (des)/notch1a*, *beamter (bea)/deltaC*, and *mind bomb* (*mib*) have been isolated in a large scale screen [24], [27], [29]–[32]. In all these mutants, the anterior somites are formed normally but the posterior somites are irregularly formed [29], [30], [33]. The zebrafish *mib2* ortholog was recently cloned [34]. The zebrafish Mib and Mib2 have common and specific Delta substrates [35] and function redundantly [34]. Three different alleles of *mib*, viz., *mib^ta52b^*, *mib^m132^* and *mib^tfi91^* with descending severity of Notch-dependent phenotypes have been characterized in our laboratory [34].

Genetic studies in mice [36] and zebrafish [37]–[40] have shown that Notch signaling is involved in pancreas development. Mice deficient in Notch signaling components, Dll1, RBP-jκ and Hes1, have an increase of endocrine pancreatic cells and depletion of progenitor cells [36], [41]. Roles of some of the Notch-related transcription factors, such as Ngn3 [42], [43], Isl1 [44], MafA [45], Pax4 and Pax6 [46], [47], in pancreas development have been demonstrated. However, the role of differential regulation of Notch signaling on pancreas development is not well understood.

Microarray is a useful method for genomewide expression profile analysis [48]. With this approach, several downstream genes of signaling pathways involved in controlling mouse pancreas development have been reported [49], [50]. Following the leads from such microarray analysis, a novel transcription factor, Myt1 [50] has been shown to be involved in pancreas development. Similarly, Mellitzer et al. also showed that a novel transcription factor, IA1, is Ngn3-regulated and required for normal differentiation of endocrine pancreatic cells [51]. High-density microarrays have been established for zebrafish and transcriptome profiles during embryogenesis have been documented [52]. In addition, new target genes involved in sonic hedgehog signaling pathway has been identified in zebrafish using microarrays [53]. However, a genomewide analysis of Notch signaling defective mutants has not been reported.

Hence, we have carried out this study with three major objectives, viz. (1) to examine the three *mib* mutant alleles for pancreas defects and understand the consequence of differential activation of Notch signaling on pancreas development using *in situ* hybridization and pancreas-specific GFP expression analysis, (2) to identify differentially regulated genes between the wt zebrafish and three different *mib* mutant alleles through microarray analysis and (3) to identify and validate the Notch-responsive genes involved in pancreas development. Through our present work, we have shown that the development of exocrine pancreas is diminished in *mib* mutants and this defect is most severe in *mib^ta52b^* followed by that in *mib^m132^* and *mib^tfi91^*. Using microarray analysis, we have identified several characterized genes, and novel genes/ESTs potentially involved in pancreas development. Validation of representative genes with real-time PCR and fluorescent double *in situ* hybridization supported the microarray data. Furthermore, this study has generated useful genomic resources identifying a number of uncharacterized ESTs/genes that may play a significant role in Notch-regulated developmental process.

## Results

### Pancreas defects in *mib* mutant alleles

**Figure 1:**
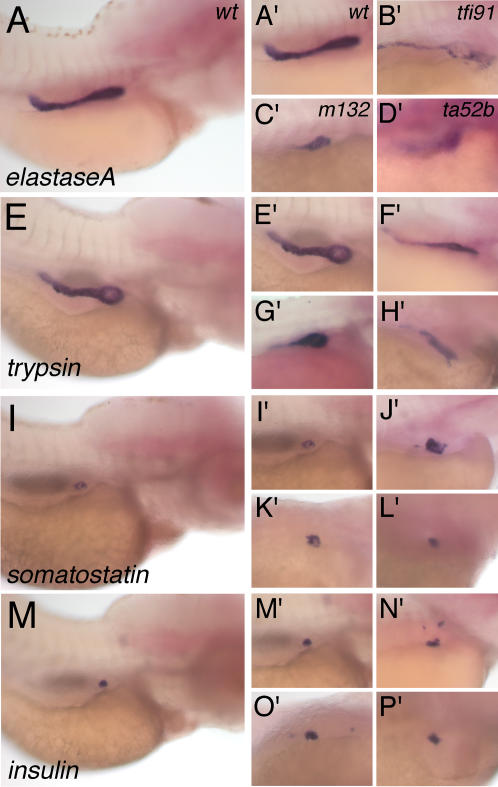
Expression pattern of pancreas-specific genes at 96 hpf in *mib* mutants and their wild type (wt) siblings. The RNA probes used for *in situ* hybridization are (A and A′–D′) *elastaseA*, (E and E′–H′) *trypsin*, (I and I′–L′) *somatostatin* and (M and M′–P′) *insulin*. The genotypes are (A, A′, E, E′, I, I′, M and M′) *wt*, (B′, F′, J′ and N′) *mib^tfi91^*, (C′, G′, K′ and O′) *mib^m132^* and (D′, H′, L′ and P′) *mib^ta52b^*. A′, E′, I′ and M′ are cropped from A, E, I and M, respectively. All panels are lateral views and anterior to the right.

**Figure 2:**
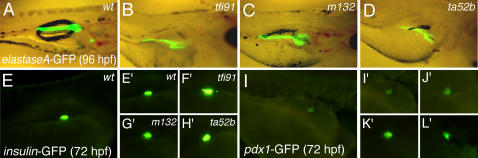
*elastaseA*-GFP, *insulin*-GFP and *pdx1*-GFP expression in embryos of *mib* mutant alleles and their wt siblings. Expression of *elastaseA*-GFP in (A) *wt*, (B) *mib^tfi91^*, (C) *mib^m132^* and (D) *mib^ta52b^* embryos was analyzed at 96 hpf. Expression of *insulin*-GFP in (E and E′) *wt*, (F′) *mib^tfi91^*, (G′) *mib^m132^* and (H′) *mib^ta52b^* embryos was analyzed at 72 hpf. Expression of *pdx1*-GFP in (I and I′) *wt*, (J′) *mib^tfi91^*, (K′) *mib^m132^* and (L′) *mib^ta52b^* embryos was analyzed at 72 hpf. Panels A–D are lateral views and the rest are dorsolateral views, oriented anterior to the right.

We carried out *in situ* hybridization for four pancreas-specific genes in *mib* mutants of 4-day post-fertilization (dpf) and their wild type (wt) siblings (Figure 1). The expression of exocrine pancreas-specific genes, *elastaseA* (Figure 1A and 1A′–1D′) and *trypsin* (Figure 1E and 1E′–H′) were diminished in *mib* mutants. In comparison to their expression in wt embryos, these two genes were highly down-regulated in *mib^ta52^* followed by those in *mib^m132^* and then *mib^tfi91^*, which are less down-regulated. This observation is in concurrence with the diminished Notch activity in these *mib* mutants [34]. We also analyzed the *elastaseA*-GFP expression in 4-dpf *mib* mutants and their wt siblings (Figure 2A–2D). The *elastaseA*-GFP expression was diminished in *mib* mutant alleles in the same order of decreasing Notch activity in these mutants, which corroborates our observation shown by *in situ* hybridization (Figure 1A and 1A′–1D′).

We further analyzed the expression of three endocrine pancreas-specific genes, *somatostatin* (δ-cell-specific), *insulin* (β-cell-specific), and *pdx1* (pancreas progenitor-specific). Levels of *somatostatin* (Figure 1I and 1I′–1L′) and *insulin* (Figure 1M and 1M′–1P′) were slightly increased in 4-dpf *mib* mutants. The *insulin*-GFP (Figure 2E and 2E′–2H′) and *pdx1*-GFP (Figure 2I and 2I′–2L′) expression analysis in *mib* mutants also showed that these genes are slightly up-regulated compared to their expression in wt embryos.

### Genomewide expression profiling

**Figure 3:**
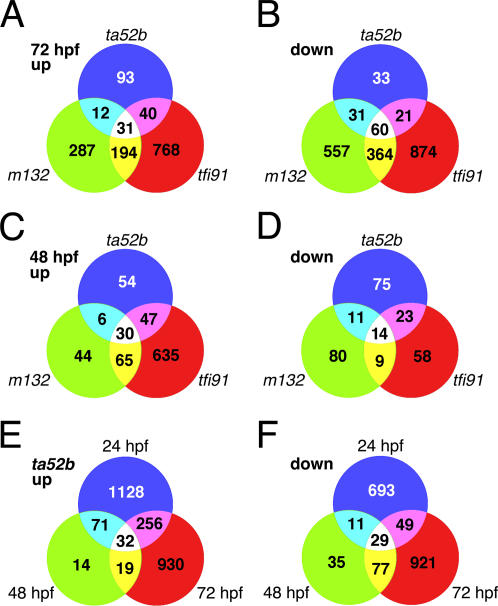
Venn diagrams show numbers of genes belonging to different groups. Differentially expressed genes specific to each *mib* mutant allele, common between two mutant alleles and common to all three mutant alleles are (A) up-regulated and (B) down-regulated at 72 hpf; and (C) up-regulated and (D) down-regulated at 48 hpf; and genes specific to each time point, common between two time points and common to all three time points are (E) up-regulated and (F) down-regulated in *mib^ta52b^* mutants. Gene set for the analysis of 72-hpf data was selected based on the criteria, q = 0.0 and score(d)>4.0; and for the analysis of 48-hpf data and the three time-point data for *mib^ta52b^* mutants were both selected based on the criterion, q = 0.0.

**Figure 4:**
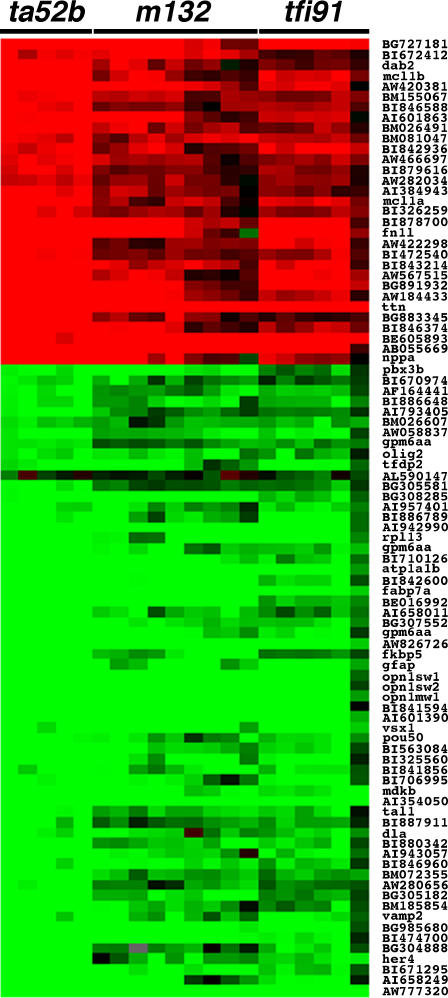
Gene expression profile of 91 differentially expressed (q = 0.0, score(d)>4.0) genes (Genbank ID) common to all three *mib* mutant alleles at 72 hpf. At least two biological repeats and two technical repeats were carried out for each mutant allele. Each horizontal strip represents expression of a single gene. Color in each cell reflects the expression level of corresponding gene in the respective sample. The up-regulation is shown in red and the down-regulation in green. Grey cells indicate the missing values. The median log2 ratio values for differential expression ranged between −4.07 and 3.32. Note: The gene symbols are mentioned instead of Genbank IDs, where the gene symbols corresponding to the Genbank IDs are available in the Zebrafish Chip Annotation Database. Three Genbank IDs, BI840762, BI839927 and BG306150, encode the same gene, *gpm6aa*.

We carried out the genomewide expression profiling of *mib* mutants at three different stages–24-, 48- and 72-hour post-fertilization (hpf). Through microarray analysis of 72-hpf embryos, we identified 1128 up-regulated and 936 down-regulated genes in *mib^ta52b^* mutants (Table S1, q = 0.0); 1464 up-regulated and 2210 down-regulated genes in *mib^m132^* mutants (Table S2, q = 0.0); and 2081 up-regulated and 2538 down-regulated genes in *mib^tfi91^* mutants (Table S3, q = 0.0). Using a PERL script, we further identified the differentially expressed genes specific to each mutant allele and common to all three *mib* mutant alleles (Figure 3). In the list of up-regulated genes, the numbers of genes specific to *mib^ta52b^*, *mib^m132^* and *mib^tfi91^* mutant alleles were 93, 287 and 768, respectively; in the list of down-regulated genes, the numbers of genes specific to *mib^ta52b^*, *mib^m132^* and *mib^tfi91^* alleles were 33, 557 and 874, respectively (Figure 3A and 3B, Table S5, S6 and S7, q = 0.0 and score(d)>4.0). The majority of these differentially expressed genes were uncharacterized genes or ESTs (Table 1). There were 91 genes common to all three *mib* mutant alleles, of which 31 were up-regulated and 60 were down-regulated (Figure 3A and 3B, Table 1). Of these 91 genes, only 27 genes were previously characterized and 64 were uncharacterized genes or ESTs (Table 1, Table S4, q = 0.0 and score(d)>4.0). The cluster tree view showed the expression profile of these 91 genes: the up-regulated ones were shown in red and the down-regulated ones in green (Figure 4). We further categorized these 91 common genes according to their known function or predicted function based on their homology to mouse and human orthologs (Table S4). Of the 27 characterized genes, there are 6 up-regulated genes, including *dab2*, *mcl1a*, *mcl1b*, *fn1l*, *ttn* and *nppa*, and 21 down-regulated genes, including *pbx3b*, *gpm6aa* (BI840762), *olig2*, *tfdp2*, *rpl13*, *gpm6aa* (BI839927), *atp1a1b*, *fabp7a*, *gpm6aa* (BG306150), *fkbp5*, *gfap*, *opn1sw1*, *opn1sw2*, *opn1mw1*, *vsx1*, *pou50*, *mdkb*, *tal1*, *dla*, *vamp2* and *her4*.

**Table 1:** Differentially expressed genes in 72-hpf *mib* mutants categorized based on their characterization status.

Gene set	Characterized genes		Uncharacterized genes/ESTs	
	Up-regulated	Down-regulated	Up-regulated	Down-regulated
*mib^ta52b^*	51	48	125	97
*mib^m132^*	72	380	452	632
*mib^tfi91^*	165	426	868	893
Genes common to all *mib* mutant alleles	6	21	25	39

Gene sets for the analysis were selected based on the criteria: q = 0.0, score(d)>4.0.

Using the same PERL script, we analyzed the 48-hpf time point data for three *mib* mutant alleles, and data of 24-hpf, 48-hpf and 72-hpf time points for the *mib^ta52b^* mutant allele. Numbers of differentially expressed genes common to all three mutant alleles or three time points, and specific to each mutant allele or time point are shown in the Venn diagram (Figure 3C–3F). At 48 hpf, there are 44 (30 up-regulated and 14 down-regulated) differentially expressed genes common to all three mutant alleles, and 129 (54 up-regulated and 75 down-regulated), 124 (44 up-regulated and 80 down-regulated) and 693 (635 up-regulated and 58 down-regulated) genes specific to *mib^ta52b^*, *mib^m132^* and *mib^tfi91^* mutant alleles, respectively (Figure 3C and 3D; Table S8, S9, S10 and S11, q = 0.0). For *mib^ta52b^* mutants, there are 61 (32 up-regulated and 29 down-regulated) genes common to all three time points, and 1821 (1128 up-regulated and 693 down-regulated), 49 (14 up-regulated and 35 down-regulated) and 1851 (930 up-regulated and 921 down-regulated) genes specific at 24 hpf, 48 hpf and 72 hpf, respectively (Figure 3E and 3F; Table S12, S13, S14 and S15, q = 0.0).

### Functional categories

**Figure 5:**
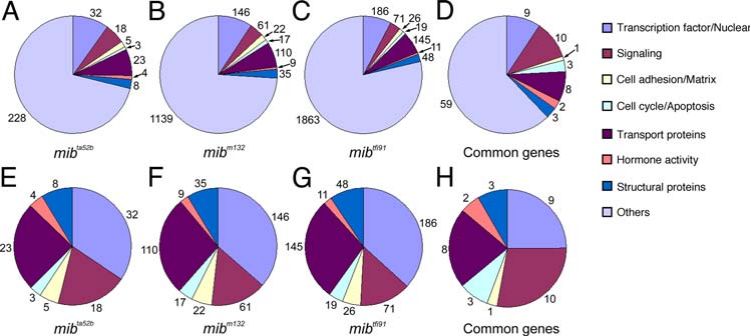
Pictorial representation of significantly up-regulated or down-regulated genes (q = 0.0, score(d)>4.0) categorized based on known/related biological functions as assigned in the zebrafish gene ontology database (Unigene Build 85). Genes in each functional group were searched by using specific or related key words appropriate for that function in the lists of differentially regulated genes for each allele, common 91 genes in all three *mib* mutant alleles, and the genes specific to each mutant allele. Absolute number of genes in each category is shown next to the corresponding pie sector. (A–D) Pie charts show numbers of genes belonging to different functional categories. (E–H) Pie charts show numbers of genes (excluding the genes without any known function) in different categories.

We used the microarray data of three *mib* mutant alleles (q = 0.0, score(d)>4.0) and the 91 common genes at 72 hpf for functional analysis. We first searched for the gene ontology and the functional similarity of their human and mouse orthologs in the Zebrafish Chip Annotation Database. Then, we classified them into different functional categories based on their known functions in zebrafish or in mouse and human orthologs, such as ‘transcription factor/nuclear’, ‘signaling’, ‘cell adhesion/matrix’, ‘cell cycle/apoptosis’, ‘transcription factors’, ‘hormone activity’ and ‘structural proteins’. Finally, we plotted them in a pie chart format either including the uncharacterized genes and ESTs (Figure 5A–5D) or excluding them (Figure 5E–5H). The majority (>70%) of the differentially expressed genes are uncharacterized genes or ESTs (Figure 5A–5D). Among the genes with known or related functions in the *mib^ta52b^* data set, about 35% belong to the category of ‘transcription factor/nuclear’ related genes and the remaining genes to other categories, such as ‘transport proteins’ (25%), ‘signaling’ (19%), ‘structural proteins’ (8.6%), ‘cell adhesion/matrix’ (5.4%), ‘hormone activity’ (4.3%) and ‘cell cycle/apoptosis’ (3.2%) (Figure 5E).

### Functional groups and pathway analysis by IPA

**Figure 6:**
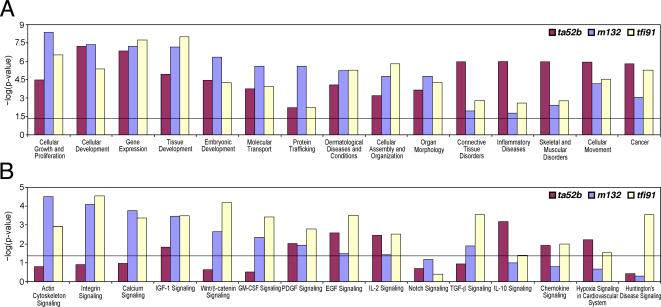
Significantly up-regulated and down-regulated genes analyzed by IPA (see Materials and Methods) for (A) enrichment of functional groups and (B) enrichment of canonical pathways, shown in histograms.

To further analyze our data set, we used IPA (see Materials and Methods) to identify functional groups and selected the top 15 ones as significantly enriched for their respective functions (Figure 6A). The significance of each function (calculated from negative log of p-value; -log 1.3 is equal to p-0.05) revealed that all the top 15 functions are highly and significantly enriched in all three *mib* alleles. Some of the significantly enriched functions are ‘cellular growth and proliferation’, ‘cellular development’, ‘gene expression’ and ‘embryonic development’. Genes related to ‘connective tissue disorders’, ‘skeletal muscular disorders’ and ‘cancer’ are significantly enriched in the *mib^ta52b^* allele.

One of the tools in IPA enables us to identify the enrichment of genes in selected canonical pathways from the input data set. The top 15 canonical pathways that are enriched in the *mib* mutants include Notch and Wnt signaling pathways (Figure 6B). Genes related to Wnt signaling were significantly enriched in *mib^m132^* and *mib^tfi91^* mutants. Notch signaling genes were enriched to a lesser extent and their enrichment was not significant. Canonical pathways, such as ‘actin cytoskeleton signaling’, ‘integrin signaling’, ‘calcium signaling’ and ‘TGF-beta signaling’, were enriched in *mib^m132^* and *mib^tfi91^*.

Notch and Wnt/β-catenin signaling were shown to be affected in *mib* mutants [27], [54], [55]. We mapped the genes that are involved in these two signaling pathways and differentially expressed in different *mib* alleles. The genes involved in Notch signaling, such as *dll1, notch1, notch2, hey1* and *herp*, were down-regulated in one, two or three *mib* alleles (Figure S1A). In Wnt/β-catenin signaling, some genes, such as *nlk, cx43*, *sox2, sox3, sox4, sox9, sox11, tcf7* and *hdac1*, were down-regulated in one or three *mib* alleles; while *sox8* and *tab1* were up-regulated (Figure S1B). Few of the genes differed in the direction of expression change in different mutant alleles. For example, *β-catenin* was up-regulated in *mib^m132^* while it was down-regulated in *mib^ta52b^* and *mib^tfi91^* (Figure S1B).

### Putative Notch signaling related genes

As positive controls of our microarray data, we searched for the known Notch signaling related genes in our list of differentially expressed genes. As expected, *heyL* was up-regulated and *dla, her4*, *her8a*, *hes5*, *hey1*, *neurod*, *neurod4*, *notch1a*, *notch2* and *notch3* were down-regulated (Table 2). To identify novel genes related to Notch signaling, we searched for the differentially expressed genes occurring at least two times in the gene sets common to all three *mib* mutant alleles at 72 hpf and 48 hpf, and the gene set common for the *mib^ta52b^* mutant allele at 72 hpf, 48 hpf and 24 hpf. We identified 31 such genes, the majority of which are novel genes or ESTs (Table S16). Of these 31 genes, only eight genes are characterized and five of them are known to be directly related to Notch signaling: *olig2* [56], *her4* [57], *hes5* (also known as *her15.1*, [58], [59]), *dla* [60] and *nort* ([61], BI886648 is 98% identical to AB259590). Moreover, *fn1l* (also known as *fn1b* or *fn3*), in conjunction with its homolog, *fn1*, has been shown to function cooperatively with Notch signaling via integrinα5 in somitogenesis [62], [63]. *gfap* and *gefiltin* are glia- and neuron-specific structural proteins respectively and, therefore, indirectly linked to Notch-modulated gliogenesis and neurogenesis [64]–[66]. These observations suggest that a certain proportion of the 23 novel genes/ESTs are very likely to be involved in or related to Notch signaling.

**Table 2:** Fold change of expression level in Notch signaling related genes at 72 hpf in three *mib* alleles.

Up-regulated genes						
Genbank ID	Gene symbol	Unigene name	Fold change in expression			
			*_mib_^ta52b^*	*_mib_^m132^*	*_mib_^tfi91^*	Average
BE016522	*heyL*	Hairy/enhancer-of-split related with YRPW motif-like	1.2068	1.4207	1.3045	1.3107

Down-regulated genes						
Genbank ID	Gene symbol	Unigene name	Fold change in expression			
			*_mib_^ta52b^*	*_mib_^m132^*	*_mib_^m132^*	Average
AI522447	*dla*	DeltaA	0.4577	0.6176	0.6013	0.5589
X97332	*her4*	Hairy-related 4	0.3598	0.6760	0.5448	0.5268
AY007990	*her8a*	Hairy-related 8a	0.5183	0.9035	0.7119	0.7112
BM072241	*hes5*	Hairy and enhancer of split 5 (*Drosophila*)	0.5338	0.6956	0.7143	0.6479
BG985516	*hey1*	Hairy/enhancer-of-split related with YRPW motif 1	0.8054	1.0734 (q = 8.9)	0.8152	0.8980
AF036148	*neurod*	Neurogenic differentiation	0.4846	0.5223	0.7155	0.5742
AF204240	*neurod4*	Neurogenic differentiation 4	0.5480	0.5452	0.6423	0.5785
X69088	*notch1a*	Notch homolog 1a	0.8032	0.5803	0.7670	0.7168
U57974	*notch2*	Notch homolog 2	1.0709 (q = 16.8)	0.7174	0.9704 (q = 12.5)	0.9196
U57975	*notch3*	Notch homolog 3	0.3863	0.5020	0.6237	0.5040

Genes were selected based on the criterion, q = 0.0. Values in brackets indicate that q values are greater than 2.0.

### Putative pancreas development related genes

We searched the gene expression data in the ZFIN database to identify the genes involved in pancreas development (Table 3, Table 4 and Table S17). There are 175 zebrafish genes in this group; however, only 98 of these genes are represented in our microarray chip. 25 of these 98 genes show significant difference (q<2.0) in gene expression in all three *mib* alleles: *cx43*, *neurod*, *ins*, *fabp2*, *cacna1c*, *zgc:92530*, *pax6b*, *try*, *olfm2*, *mt2*, *xbp1*, *hsd11b2*, *zgc:92292*, *gadd45a*, *wu:fb59c09*, *cbs*, *tdh*, *gstp1*, *ssr4*, *gatm*, *rims2*, *atp1a3a*, *isl1*, *hoxc8a* and *notch1a* (Table 3). The rest are differentially expressed either in one or two of these three alleles (Table S17). In addition, we identified 5 genes, namely *gad1* [67], *igfbp2* [68], *zgc:112198* [69], *wu:fc21h08* [70] and *isl3* [71] based on the available functional analysis reports for their mouse orthologs, and 4 other genes, namely *sfrs5*, *zgc:56374*, AW281193 and BI846588 based on their gene ontology information available in the zebrafish gene annotation database (Table 4). These 9 genes are likely to play a role in zebrafish pancreas development.

**Table 3 pone-0001479-t003:** Expression level of differentially expressed genes (q<2.0) involved in pancreas development at 72 hpf in three *mib* alleles shown in terms of fold change.

Down-regulated genes						
Genbank ID	Gene symbol	Unigene Name	*mib^ta52b^*	*mib^m132^*	*mib^tfi91^*	Average fold change
AF035481	cx43	Gap junction protein, alpha 1	0.7771	0.5925	0.8182	0.7293
AF036148	neurod	Neurogenic differentiation	0.4846	0.5223	0.7155	0.5741
AF180921	fabp2	Fatty acid binding protein 2, intestinal	0.6514	0.7622	0.5752	0.6630
AI601297	pax6b	Paired box gene 6b	0.7366	0.7788	0.8862	0.8005
AJ297822	try	Trypsin	0.8176	0.8766	0.8712	0.8551
AW115690	olfm2	Olfactomedin 2	0.6421	0.5667	0.8625	0.6904
BG882996	hsd11b2	Hydroxysteroid 11-beta dehydrogenase 2	0.8263	0.9139	0.7796	0.8400
BI896418	gatm	Glycine amidinotransferase	0.6641	0.8305	0.6792	0.7246
BI981058	rims2	Regulating synaptic membrane exocytosis 2	0.5887	0.7021	0.9263	0.7390
BM183338	atp1a3a	ATPase, Na+/K+ transporting, alpha 3a polypeptide	0.7161	0.7431	0.8743	0.7778
D21135	isl1	Islet1	0.4843	0.8373	0.7885	0.7033
Y14544	hoxc8a	Homeo box C8a	0.8644	0.5562	0.7260	0.7155
AI497360	zgc:92530	Zgc:92530	1.1962	0.8602	0.8983	0.9849
AW184187	mt2	Metallothionein 2	1.3597	0.7799	0.8513	0.9970
BI709417	wu:fb59c09	Wu:fb59c09	1.2429	0.6583	0.8936	0.9316
BI882972	gstp1	Glutathione S-transferase pi	0.8352	0.7378	1.1683	0.9138
BI885968	ssr4	Signal sequence receptor, delta	1.2889	0.4576	0.7009	0.8158
X69088	notch1a	Notch homolog 1a	0.8032	0.5803	0.7670	0.7168

Up-regulated genes						
Genbank ID	Gene symbol	Unigene Name	*mib^ta52b^*	*mib^m132^*	*mib^tfi91^*	Average fold change
AF305689	cacna1c	Calcium channel, voltage-dependent, L type, alpha 1C subunit	1.1653	1.1483	1.0988	1.1375
BI672019	gadd45a	Growth arrest and DNA-damage-inducible, alpha	1.1878	1.1591	1.0964	1.1478
BI879550	cbs	Cystathionine-beta-synthase	1.6677	1.3112	1.2058	1.3949
AF036326	ins	Preproinsulin	1.3530	0.8345	0.8377	1.0084
AY029577	xbp1	X-box binding protein 1	1.3598	0.6465	1.1352	1.0471
BI476180	zgc:92292	Zgc:92292	0.8291	1.1458	1.1390	1.0380
BI882450	tdh	L-threonine dehydrogenase	1.8600	1.0871	0.8719	1.2730

**Table 4:** Expression level of differentially expressed genes (q<2.0), predicted to be involved in pancreas development based on functional homology, at 72 hpf in three *mib* alleles shown in terms of fold change.

Down-regulated genes						
Genbank ID	Gene symbol	Unigene Name	*mib^ta52b^*	*mib^m132^*	*mib^tfi91^*	Average fold change
AF017266	gad1	Glutamate decarboxylase 1	0.6482	1.0506	0.6659	0.7882
AF198033	igfbp2	Insulin-like growth factor binding protein 2	0.9955	0.6396	0.7825	0.8059
BI887742	sfrs5	Splicing factor, arginine/serine-rich 5	0.9756	0.4959	0.8615	0.7777
BI533195	wu:fc21h08	Wu:fc21h08	1.3516	0.7193	0.9067	0.9925
BI891976	zgc:56374	Zgc:56374	0.8668	0.6375	0.8464	0.7836
AW281193		Transcribed locus	0.9340	0.7479	0.8379	0.8399
AW282071	zgc:112198	Zgc:112198	0.3908	0.6969	1.2720	0.7866

Up-regulated genes						
Genbank ID	Gene symbol	Unigene Name	*mib^ta52b^*	*mib^m132^*	*mib^tfi91^*	Average fold change
D38454	isl3	Islet3	0.8717	1.2938	1.1984	1.1213
BI846588		Transcribed locus, weakly similar to NP_002572.2 pregnancy-associated plasma protein A preproprotein; insulin-like growth factor-dependent IGF binding protein-4 protease; aspecific BCL2 ARE-binding protein 2 [Homo sapiens]	2.0230	1.3549	1.5408	1.6396

### Fluorescent double *in situ* hybridization validation of pancreas development related genes

**Figure 7:**
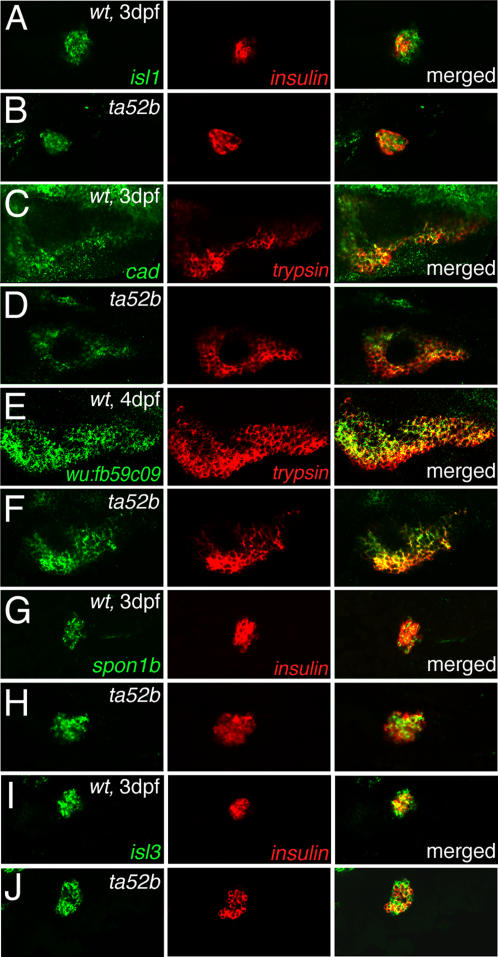
Validation of expression profile of differentially expressed genes with fluorescent double *in situ* hybridization. Expression of down-regulated genes: (A and B) *isl1*, (C and D) *cad* and (E and F) *wu:fb59c09* and up-regulated genes: (G and H) *spon1b* and (I and J) *isl3* in (B, D, F, H and J) *mib^ta52b^* mutants embryos and (A, C, E, G and I) wild-type embryos. Left panels show respective gene expression in green; middle panels show *insulin* (endocrine) or *trypsin* (exocrine) expression in red; right panels show merged pictures. Expression of *wu:fb59c09* is shown in 4-dpf embryos, and the rest are in 3-dpf embryos. All embryos are anterior to the left and ventral views.

To validate the microarray data for the genes predicted to be related to or involved in pancreas development, we carried out fluorescent double *in situ* hybridization at 3-dpf embryos for five down-regulated genes: *trypsin*, *isl1*, *cad*, *wu:fb59c09* and *notch1a*, and five up-regulated genes: *insulin*, *isl3*, *spon1b*, *glo1* and *txnip* (Table 3, Table 4 and Table S17). Exocrine *trypsin* and endocrine *insulin* are predictably down- and up-regulated, respectively (Figure 7). However, *notch1a*, *glo1* and *txnip* were not detected in the pancreas (see Discussion).

#### Down-regulated genes

*isl1* encodes a zebrafish insulin gene enhancer binding protein and it has 81% identity to mouse LIM/homeodomain transcription factor ISL1 (islet-1). *isl1* was expressed in nervous system, liver (data not shown) and pancreas of 3-dpf embryos. Our result is similar to that in earlier studies, which showed that *isl1* is expressed in organs such as nervous system, liver and pancreas at various developmental stages of zebrafish [58], [72]–[77]. Its expression was strongly diminished in the nervous system (data not shown) of *mib^ta52b^* mutants; however, its expression was only slightly reduced in the endocrine pancreas compared to wt embryos (Figure 7A and 7B). Our microarray results also showed that *isl1* is down-regulated in *mib^ta52b^* mutants (Table 3).

*cad* encodes zebrafish carbamoyl-phosphate synthetase 2. Microarray analysis showed that *cad* is down-regulated (Table S17). *cad* was expressed in endodermal organs, such as pancreas, intestine and liver, which is similar to that observed in earlier reports [78], [79]. In *mib^ta52b^* mutants, *cad* expression was down-regulated in all these tissues (Figure 7C and 7D; data not shown).

*wu:fb59c09* is a zebrafish EST with significant similarity to a hypothetical gene, LOC570477. The human ortholog of this gene is *Peroxiredoxin 4* (*prx4*). *In situ* hybridization results showed that this gene is expressed in liver, pancreas, intestine and other endodermal tissues (data not shown). It was down-regulated in both endocrine and exocrine pancreas of *mib^ta52b^* mutants compared to that in their wt siblings (Figure 7E and 7F).

#### Up-regulated genes

*spon1b* encodes an extracellular matrix protein, whose C-terminal contains five repeats identified previously in Thrombospondin and other proteins implicating in cell adhesion. Fluorescent double *in situ* hybridization results showed that this gene is expressed in endocrine pancreas, similar to earlier studies [77], [79], and was up-regulated in *mib^ta52b^* mutants compared to that in their wt siblings (Figure 7G and 7H).

*isl3* encodes a zebrafish insulin gene enhancer binding protein homolog. It has high similarity to human ISL1 transcription factor, LIM/homeodomain (Islet-1) and ISL2 transcription factor, LIM/homeodomain (Islet-2). *isl3* was expressed in endocrine pancreas, liver and intestine and its expression was up-regulated in *mib^ta52b^* mutants (Figure 7I and 7J; data not shown).

### Real-time PCR validation of global expression profile

To further validate the microarray expression profile analysis, we carried out real-time PCR for 25 genes using the primers listed in Table S18. All the PCRs yielded the expected 300 base pair products. The real-time PCR ratios are basically similar to those of microarray data (Table 5), which confirms the validity of the array data.

**Table 5:** Real-time PCR validation of microarray data.

A. Down-regulated genes				
Genbank ID	Unigene Name	Gene symbol	Microarray fold change ratio	Real-time PCR ratio
AJ297822	Trypsin	try	0.81	0.18
D21135	Islet1	isl1	0.48	0.17
X97332	Hairy-related 4	her4	0.35	0.04
U57975	Notch homolog 3	notch3	0.38	0.12
AI522447	DeltaA	dla	0.45	0.22
X69088	Notch homolog 1a	notch1a	0.80	0.22
AF109373	Opsin 1 (cone pigments), short-wave-sensitive 1	opn1sw1	0.14	0.004
AF017266	Glutamate decarboxylase 1	gad1	0.65	0.25
BI891976	Zgc:56374	zgc:56374	0.86	1.15
AY583322	elastase A		0.89	0.07
AW019843	Somatostatin 1	sst1	0.62	0.13
AW282071	Zgc:112198	zgc:112198	0.39	0.10
AW281193	similar to rat insulinoma gene		0.93	0.49
BI887742	Splicing factor, arginine/serine-rich 5	sfrs5	0.97	0.87
AF198033	Insulin-like growth factor binding protein 2	igfbp2	0.99	0.13
BI709417	Wu:fb59c09 (Danio rerio similar to Peroxiredoxin 4)	prx4	0.93¶	0.88

B. Up-regulated genes				
Genbank ID	Unigene Name	Gene symbol	Microarray fold change ratio	Real-time PCR ratio
AB062116	Heat shock cognate 70-kd protein	hsp70	5.14	11.55
AF036326	Preproinsulin	ins	1.35	1.22
BM183964	Glyoxalase 1	glo1	1.40	1.08
AF036325	Insulin promoter factor 1, homeodomain transcription factor	ipf1	1.18	0.20
BI890045	PERP, TP53 apoptosis effector	perp	1.66	0.70
BI846588	Transcribed locus, weakly similar to insulin-like growth factor-dependent IGF binding protein-4 protease		2.02	0.48
BI533195	Wu:fc21h08 (Danio rerio insulin-like growth factor 2 precursor)	igf2	1.35	0.86
D38454	Islet3	isl3	1.12¶	0.19
AB006087	Spondin 1b	spon1b	1.28	0.36

The real-time PCR ratio in *mib^ta52b^* allele at 72 hpf is compared with the microarray data expressed as fold change values. The Genbank ID, Unigene name and Gene symbol were obtained using the Zebrafish Chip Annotation Database, (Unigene Build 85), http://giscompute.gis.a-star.edu.sg/govind/zebrafish/version2/.

¶ Average microarray ratio of three *mib* mutants.

We noticed that the results from two methods for few genes are not consistent. Therefore, to further address this question statistically, we evaluated the linear relationship between the microarray ratio (fold change) and the real-time PCR ratio for *mib^ta52b^* allele at 72 hpf using Pearson's correlation coefficient. It indicates a statistically significant positive correlation between these two sets of ratios (R = 0.914, R^2^ = 0.835, p<0.001). This shows that the two sets of ratios are significantly (positively) correlated and the data are acceptably reliable [80].

## Discussion

*mind bomb* mutants have compromised Notch activity due to mutations in the *mib* gene and serve as a unique resource to study the role of Notch signaling on various developmental processes [27], [29]. Moreover, there are several *mib* alleles with different genetic severity available [34]. In this study, we have examined the *mib* mutants for defects in pancreas development using *in situ* hybridization and pancreas-specific GFP expression analyses, and compared the global expression profile of three *mib* mutant alleles and their wt siblings using oligo microarray chips.

### *mib* alleles are unique for studying Notch signaling

The *mib* mutants have diminished exocrine pancreas development as evidenced by decrease in *elastaseA* and *trypsin* expression. The *mib^ta52b^* mutant allele showed maximal decrease in exocrine pancreas followed by *mib^m132^* and *mib^tfi91^* alleles. This indicates that there is a dose-dependent response of Notch signaling on pancreas development, since these alleles have different degree of compromised Notch activity [34]. Our GFP expression analysis in *elastaseA*-GFP lines also showed a similar effect of graded Notch signaling on pancreas development. Likewise, based on *in situ* hybridization results for exocrine pancreas-specific genes, such as *mnr2a*, *ptf1a* and *trypsin* in *mib^ta52b^* mutants and DAPT-treated *mib^ta52b^* mutants, Zecchin *et al*. concluded that a blockage of Notch signaling decreases the number of exocrine pancreatic cells [40]. Earlier study by Esni *et al*. also showed the role of Notch signaling in exocrine pancreas development of zebrafish [37].

The *in situ* hybridization results for the endocrine pancreas-specific genes, such as *insulin* and *somatostatin*, showed slight increase in their expression in *mib* mutant alleles; however, a dose-dependent response of Notch signaling was not obvious (Figure 1). The GFP expression analyses in *insulin*-GFP and *pdx1*-GFP lines also showed a slight increase in endocrine pancreas in *mib* mutant alleles (Figure 2). Similar to our results, Zecchin *et al*. also showed that in *mib* mutants (*mib^ta52b^*) there is an increased expression of *insulin* and *somatostatin* [40].

### Microarray analysis in *mib* alleles

In this study, we have used the microarray chips with 16,416 probes, representing 15,800 unique zebrafish genes. Earlier studies have successfully used this version of microarray chips for elucidating genes involved in zebrafish embryogenesis [52], liver tumor progression [81] and Hedgehog signaling [53]. Comparison of our microarray results for three *mib* mutant alleles at 72 hpf showed that the number of differentially expressed genes (q = 0.0, score(d)>4.0) in *mib^tfi91^* allele (2352) is greater than that in *mib^m132^* (1536) and *mib^ta52b^* (321) alleles (Figure 3A and 3B). Interestingly, this is inversely related to the severity of their phenotypes, such as fused somite borders, diminished tail pigmentation, neuronal hyperplasia and diminished *her4* expression: most severe in *mib^ta52b^* allele followed by those in *mib^m132^* and *mib^tfi91^* alleles [34]. So far, there is no known molecular mechanism to explain this finding. Future studies need to be focused in this direction.

We observed fewer differentially expressed genes at 48 hpf compared to that at 72 hpf for all three *mib* mutant alleles. This is in concurrence with the phenotypes of these three *mib* alleles, which are less obvious at 48 hpf compared to that at 72 hpf [34]. Owing to the large number of differentially expressed genes at these two time points, we narrowed our focus on the 72-hpf data set alone. In all three *mib* mutant alleles at 72 hpf, the majority (>70%) of the differentially expressed genes are uncharacterized genes or ESTs. This is because the zebrafish genome annotation has not been completed and the majority of genes remain as uncharacterized ESTs. Though a number of differentially expressed genes have been identified as full-length clones with ZGC IDs, they still remain unannotated. Gene Ontology (GO) analysis of differentially expressed genes of *mib^ta52b^* allele showed that less than 29% of the genes are characterized, the majority (35%) of which belong to the category of ‘transcription factors/nuclear’ followed by the category of ‘signaling molecules’ (19%). This could be due to the fact that the *mib* mutants are defective in Notch signaling, which is one of the fundamental signaling pathways required for proper development of an organism.

### Genes potentially involved in Notch signaling

Our microarray data is reliable, because some expected Notch signaling related genes did appear in the transcriptome, such as up-regulated *heyL* and down-regulated *dla*. Comparison of three sets of genes (72 hpf and 48 hpf for three *mib* alleles and 24 hpf, 48 hpf and 72 hpf of the *mib^ta52b^* allele) showed that there are 31 genes common to all three gene sets (Table S16). Five of these 31 genes, namely *olig2* [56], *her4* [57], *hes5* (also known as *her15.1*, [58], [59]), *dla* [60] and *nort* [61], are previously shown to be involved in Notch signaling. Particularly, based on similar global expression analyses of *notch1a* and *notch3* morpholino morphants, Tsutsumi and Itoh showed that *nort* is a putative noncoding RNA regulated by Notch signaling in zebrafish [61]. Therefore, it is likely that the rest of the genes in this group are a part of the Notch connected network and hence serve as a useful resource to further identify novel genes working downstream of Notch.

### Genes potentially involved in pancreas development

As a major focus of our current study, we searched for the genes related to pancreas development. As of now, there is no bioinformatic tool available to classify the zebrafish genes according to their biological functions. Therefore, we carried out a detailed manual search for the genes related to pancreas development. Using the ZFIN *in situ* hybridization expression database, we found 98 zebrafish genes that have been previously shown to be expressed in pancreas (Table 3, Table 4 and Table S17). The microarray expression profile for these genes showed that their expression profile is significantly different (q<2.0) in at least one of the three *mib* alleles. Several genes, such as *neurod*, *isl1* and *pax6b* [82]–[84] were formerly shown to be responsive to Notch signaling in mice.

However, the fold change of expression for each gene is different in these three alleles. Only 25 out of these 98 zebrafish genes involved in pancreas development show significant difference (q<2.0) in expression profile in all three *mib* alleles. Out of these 25 genes, only 15 genes show consistent up-regulation (3 genes) or down-regulation (12 genes) in all three alleles. Apart from these 98 genes, we discovered 9 differentially expressed genes (Table S19), which are likely to play a role in zebrafish pancreas development based on their functional homology to mouse and human orthologs. It is definitely worth addressing in the future.

### Is Mib Notch-specific?

So far, the experiments have unequivocally proved that Mib is an essential component of Notch signaling: it ubiquitylates and then endocytoses Delta with the extracellular part of Notch and therefore allows the intracellular part of Notch to enter the nucleus in the receiving cell and activate downstream target genes [27]. There is also evidence to suggest that Mib may be linked to Wnt signaling. Riley, et al. showed that heat shock-driven *wnt1* expression in *mib* mutants leads to a partial rescue of its hindbrain metameric patterning phenotype [54]. Furthermore, knockdown of *wnt3a* and *wnt8b* in *Dfw5* mutants, where *wnt1* and *wnt10b* are deleted, resulted in the lost of boundary cells in hindbrain, which is similar to that in *mib^ta52b^* mutants [54]. However, this is in sharp contrast to *wnt1* and *tcf3b* morphants, where the boundary cells are increased [55]. Therefore, the mechanism remains to be determined.

From our IPA pathway analysis in all three *mib* alleles, we found enrichment of several differentially expressed genes belonging to various canonical pathways that have not been shown to directly link to Mib, including IGF-1, PDGF, EGF and IL-2 signaling pathways (Figure 6B). This raises the possibility that Mib may be somehow involved in these signaling pathways. Furthermore, mouse DAPK and zebrafish Jagged2a have been shown to be substrates of Mib E3 ligase [85], [86]. With a yeast two-hybrid screen, we also found that Mib binds to proteins involved in endocytosis and the ubiquitin-proteasome pathway (Chengjin Zhang, Jason Kin Wai Koo, Qing Li, Haoying Xu and Y.-J. J., unpublished data). Similarly, Snx5 has been identified as a Mib-binding protein from a yeast two-hybrid screen, which is colocalized with Mib in early endosomes and required for hematopoiesis and vasculogenesis [87]. All these observations suggest that Mib may not be Notch-specific and can also work with other pathways, just as previously shown for another E3 ligase, Itch, which targets Notch receptor and links to TNF through JNK [88]–[90]. Alternatively, the gene expression change could simply reflect the tissue/organ defects that are inflicted by a failure in Notch signaling. Our pathway analysis on microarray data supplies a good resource for examining whether Mib functions beyond Notch signaling and/or for testing what genes are involved in the tissue/organ mis-patterning caused by compromised Notch activity.

### Validation of microarray data

To validate the microarray expression profile, we carried out fluorescent double *in situ* hybridization for ten genes (5 down-regulated and 5 up-regulated) and real-time PCR for 25 genes. Out of these ten genes, *insulin*, *trypsin*, *spon1b*, *cad*, *isl1*, *wu:fb59c09*, *notch1a*, *txnip* and *glo1* are previously shown to be involved in the development of and/or expressed in zebrafish pancreas; and *isl3* is predicted to be related to pancreas development.

The microarray expression profile of *trypsin* and *isl1* showed a down-regulation in *mib^ta52b^* allele and this was validated by our fluorescent double *in situ* hybridization and real-time PCR (Figure 7 and Table 5). *wu:fb59c09* was slightly up-regulated (1.24) in microarrays of *mib^ta52b^* allele but it was down-regulated in *mib^m132^* and *mib^tfi91^* alleles (Table 3). However, our fluorescent double *in situ* hybridization and real-time PCR validations showed that it is down-regulated in the *mib^ta52b^* allele (Figure 7 and Table 5). *cad* expression was decreased in the *mib^ta52b^* allele by double *in situ*, which is consistent with the microarray data (Figure 7 and Table S17). Earlier studies have shown that all these four genes are expressed in zebrafish pancreas at various developmental stages [37], [75], [78], [79]. However, the role of Notch signaling on their expression profile in pancreas is not known except *isl1*. *isl1* was shown to be up-regulated in primary neurons of *mib* mutants at 16s to 20s stage [91], but the effect of Notch signaling on its expression at later stages has not been studied. Furthermore, no observation has been made on its expression in pancreas. In contrast, our *in situ* hybridization results in 3-dpf embryos showed that the expression of *isl1* in *mib^ta52b^* allele is down-regulated in nervous system but its expression in pancreas is only slightly reduced compared to that in wt embryos. In support of this observation, our microarray and real-time PCR results also showed that *isl1* is down-regulated in *mib^ta52b^* mutants (Table 3 and 5).

Two up-regulated genes, *insulin* and *spon1b*, were validated. The microarray data showed that these two genes are up-regulated in the *mib^ta52b^* allele and this is supported by our fluorescent double *in situ* hybridization. *isl3* was slightly increased by fluorescent double *in situ* hybridization. However, it was consistently decreased in *mib^ta52b^* allele by microarray analysis and real-time PCR (Figure 7, Table 4 and 5).

In contrast to the available information from ZFIN, which shows the gene expression pattern mainly up to 2 dpf, we did not detect *notch1a*, *glo1* and *txnip* expression in the pancreas using fluorescent double *in situ* hybridization at 3 dpf, though we did detect *notch1a* expressed in, for example, hindbrain. It could be due to the stage difference or technical reasons. However, our real-time PCR results validated microarray expression for *notch1a* and *glo1* (Table 5).

The ratio comparison between microarray and real-time PCR for genes, such as *ipf1*, *perp*, *igf2*, *isl3*, *spon1b* and BI846588, showed that there is slight up-regulation in microarray, but down-regulation in real-time PCR. However, it is evident that the overall expression alteration of all 25 genes is statistically comparable, although the actual fold change values in real-time PCR and microarray are not always commensurable. Such variation in values is likely due to the difference in sensitivity [80]. Nevertheless, our *in situ* hybridization results are highly consistent with our microarray profile.

In conclusion, the microarray analyses carried out in this study provide a useful resource of global gene expression profile of *mib* mutants defective in Notch signaling. Functional analysis of differentially expressed genes will shed light on their role in Notch signaling and various developmental processes.

## Materials and Methods

### Zebrafish wild type embryos and *mind bomb* mutants

We used AB strain wild type (wt) and three different *mib* alleles of different genetic severity, viz., *mib^ta52b^*, and *mib^m132^*, *mib^tfi91^* [34]. *mib^ta52b^* carries a missense mutation (M1013R) in the C-terminal-most RING finger domain; *mib^m132^* carries a nonsense mutation (C785stop) leading to a truncated protein and *mib^tfi91^* contains a nonsense mutation (Y60stop) [27]. *mib^ta52b^* and *mib^m132^* are strong and weak antimorphic alleles, respectively, whereas *mib^tfi91^* is a null allele [34]. All animal procedures were approved by the Biological Resource Centre, A*STAR.

### Fish maintenance and sample collection

Fish were maintained in the IMCB zebrafish facility according to standard procedures. Crosses were set up in the evening and the barrier was lifted in the next morning. After half an hour, the fertilized embryos were collected and maintained at 28.5°C in egg water supplemented with methylene blue. For microarray analyses, the wt embryos were collected at 24 hpf, 48 hpf and 72 hpf, snap-frozen in liquid nitrogen and stored at -80°C. Mutants were separated from their wt siblings and frozen stored in the same way. At least two independent biological replicates were taken for each sample. All the samples were collected from the same cohort of fish to maintain a uniform genetic background.

For whole mount *in situ* hybridization and GFP analysis, the fertilized embryos were collected and grown at 28.5°C. Embryos were transferred to egg water with 0.033% phenylthiourea (PTU), which inhibits pigmentation, after 12 hpf and fixed/collected at appropriate stages.

### *In situ* hybridization and fluorescent double *in situ* hybridization

DNA clones for making *in situ* hybridization probes were obtained from the Expressed Sequence Tag (EST) clone collection at the Genome Institute of Singapore (GIS) and the Institute of Molecular and Cell Biology (IMCB). Whole mount *in situ* hybridization using digoxigenin (DIG) (Roche) labeled RNA probes was carried out as previously reported [92]. Goat anti-DIG antibody conjugated to alkaline phosphatase (AP) was used for probe detection and NBT-BCIP was used as the substrate for color development. *In situ* hybridized embryos were observed using light microscope and the photos were taken with Zeiss Imager M1 microscope.

DIG- and fluorescein-labeled probes were generated via standard protocols. Embryos were proceeded for fluorescent double *in situ* hybridization with protocols previously reported [32], [86]. However, the incubation temperature for probes was changed to 60°C to reduce the background. Photos were taken with Olympus Fluoview FV1000 microscope.

### GFP expression analysis

To screen for defects in pancreas development, we have used three pancreas-specific GFP transgenic lines, viz., *elastaseA*-GFP (*elaA*-GFP) [93], *insulin*-GFP and *pdx1*-GFP lines [94]. These GFP lines were crossed with the heterozygous *mib* mutants and the offspring (F1) were grown until maturity. The F1 siblings carrying *mib* mutations were intercrossed to obtain embryos for GFP expression analysis. Photos were taken with Leica MZ FLIII microscope.

### RNA extraction

Total RNA from the frozen embryos was extracted with Trizol (Gibco BRL) and cleaned with the Qiagen RNeasy mini kit. RNA quality was determined by gel electrophoresis, and the concentration was measured with a UV spectrophotometer. To reduce the bias, we used a common reference RNA for each time point, which was prepared at one time by extracting RNA from stage-matched wild type embryos. The RNA extracts were stored at −80°C.

### Microarray construction, target preparation and hybridization

The zebrafish microarrays were printed at GIS [52]. Oligonucleotide probes for this array were designed by Compugen (USA) and synthesized by Sigma Genesis (USA). For each gene, one 65-mer-oligonucleotide probe was designed from the 3′ region sequence. Each probe was selected from a sequence segment that is common to a maximum number of splice variants predicted for each gene. The arrays contained 16,416 probes, representing 15, 800 unique zebrafish genes (UniGene build 85). In addition, the arrays also contained 170 spots representing *β-actin* gene probes as controls. The probes were suspended at a concentration of 20 µM in 3X SSC and spotted onto poly-L-Lysine coated microscope slides using custom-built DNA microarrayer.

For fluorescent labeling of target cDNAs, 20 µg of total RNA (10 µg, when the RNA quantity was limited) from reference and experimental samples were reverse-transcribed in the presence of Cy3-dUTP and Cy5-dUTP (Amersham Biosciences), respectively. Labeled target cDNAs were combined, concentrated and resuspended in DIG EasyHyb (Roche). Hybridizations on microarray slides were performed at 42°C for 16 h using MAUI Mixer FL (BioMicro Systems) as explained earlier [52], [95]. At least two independent biological replicates were taken for each sample. At least two to three independent replicate hybridizations (technical repeats) were performed for each biological repeat sample (Table S20).

### Scanning, filtering and data normalization

The arrays were scanned by the GenePix 4000B microarray scanner (Axon Instruments) to generate 16-bit TIFF image files. GenePix Pro 4.0 image analysis software (Axon Instruments) was used to measure the fluorescent signal intensity of the array features and local background on TIFF images. Only the gene features with signal background ratio more than 1.5 were used for analysis. The 16-bit TIFF image files and the gpr files with Cy3 and Cy5 signal intensities were uploaded into the GIS-developed Microarray Database (mAdb). Median normalization of the sample and reference channel intensity values was performed using the intensity-based log ratio median method [96]. The extracted intensity data from the mAdb database were normalized by Lowess normalization method and analyzed by modified *t*-statistic Significance Analysis of Microarrays (SAM) [97]. The microarray data files have been submitted to the Gene Expression Omnibus (GEO) and the accession number is GSE8522.

### Gene annotation

The gene annotations were carried out by using the Zebrafish Chip Annotation Database (MySqL) (UniGene build 85) (http://giscompute.gis.a-star.edu.sg/govind/zebrafish/version2) developed and maintained by GIS. This database contains putative annotations for the probes in the zebrafish oligonucleotide array. We queried this database with the Genbank ID to obtain the following information: (1) Compugen description, (2) Zebrafish UniGene ID (build 85), (3) Zebrafish UniGene description (build 85), (4) Entrez Gene description, (5) Entrez Gene ID and Gene symbol, (6) GO term, (7) Locus Link, (8) UniGene protein similarity and description (mouse and human), (9) Full-length or assembled sequences, (10) HomoloGene (human and mouse, build 38.1) and (11) chromosomal location of the gene. Full-length sequences or the longest available gene sequences were obtained from the NCBI UniGene database (http://www.ncbi.nlm.nih.gov/sites/entrezdbunigenecmd). The Genbank IDs are referred to as ‘genes’ throughout this article.

### Microarray data analysis

We applied Significance Analysis of Microarrays (SAM) [97] to identify statistically significant genes in each case. Since we have different number of replicates for different alleles, we used different thresholds (q = 0.0, score(d)>4.0 [this represents that the absolute value of score(d) is greater than 4.0, namely, |score(d)|>4.0] at 72 hpf; q = 0.0 at 48 hpf; q = 0.0 at 24 hpf) to select similar number of genes. However, the thresholds we used are all stringent (in every case the q-value is less than 2) and hence the false discovery rate (FDR, value expressed in %) in each case does not exceed 5. The FDR indicates the outcome with which the gene selected to be differentially expressed by the SAM analysis is likely to be occurring by chance. The score(d) indicates a statistic parameter, which is numerator(r) divided by denominator(s+s0) and hence serves as a cut-off point along with the q value. The numerator(r) value indicates the actual gene expression change shown as log2 value.

For the analysis of 72-hpf time point data, we selected those genes with q = 0.0 and scored(d)>4.0 from the SAM generated data for all three *mib* alleles (Table S1, S2 and S3). The description for all the gene sets was obtained from the Zebrafish Chip Annotation Database [52]. Based on the most recently available information of the zebrafish gene annotation, these gene sets were classified as characterized and uncharacterized genes (Table 1). Furthermore, we manually searched for two sets of genes: one with functions related to Notch signaling and the other with functions related to pancreas development (Table 2–4). The expression profile values (log2) for the genes involved in the Notch pathway and pancreas development were obtained from the SAM analyzed data set. The *in situ* hybridization gene expression data for the genes related to pancreas development were obtained from the ZFIN database (http://zfin.org/cgi-bin/webdriverMIvalaa-xpatselect.apg). If the gene was previously shown to be involved in pancreas development, we classified them as the ‘genes involved in pancreas development’. If there is no functional relationship to zebrafish pancreas development but only functional homology to the human or mouse genes related to pancreas development, we classified them as the ‘genes predicted to be involved in pancreas development’.

A PERL script was used to identify the differentially expressed (q = 0.0, score(d)>4.0) genes (Genbank IDs) that are common among all three *mib* alleles (Table S4), between two different alleles and specific to each allele (Table S5, S6 and S7) at 72 hpf, and to remove duplicate genes (Genbank IDs), if any. Using this method, a group of 91 genes common to all three *mib* alleles were classified based on their function and characterization status (Table S4). These 91 genes were hierarchically clustered with TreeView_vers_1_60 software [98] and tree view image was generated using Adobe Illustrator (Fig. 4).

For the analysis of 48-hpf time point data, the gene sets with q = 0.0 were used. The same PERL script was also used here to find the common genes and the allele-specific genes (Table S8, S9, S10 and S11). The SAM data (q = 0.0) for the *mib^ta52b^* on all three time points (24 hpf, 48 hpf and 72 hpf) were analyzed and queried using the PERL script to find out the gene sets that are common to all time points and specific to each time point (Table S12, S13, S14 and S15).

### Functional groups and pathway analysis

Differentially expressed genes of three *mib* mutant alleles were subjected to Ingenuity Pathways Analysis (IPA) to identify the enrichment of genes in specific functional groups and pathways (IPA, Version 4, Ingenuity® Systems, http://www.ingenuity.com). The IPA accepts human UniGene IDs as one of the identifiers for data upload and analysis. For this reason, the differentially expressed genes of *mib* mutants were mapped to their human homologs using the HomoloGene database and zebrafish UniGene mapping tool established at the GIS (http://giscompute.gis.a-star.edu.sg/govind/unigene_db/). Human homologs of up- and down-regulated genes of the mutants were analyzed by using IPA tools and the enrichment of functional categories and canonical pathways with reference to the Ingenuity Pathways Knowledge Base (IPKB) were documented. Initially, differentially expressed genes of the three mutant alleles were individually analyzed. Subsequently, the enrichment patterns were compared among the mutants to identify the conservation of functional groups among the mutants.

Using the input data set (human homologs of zebrafish genes differentially expressed in the *mib* mutants), IPA identified a set of genes that are enriched for a particular function or pathway and the enrichment is represented as ratio. The ratio refers to the number of input genes associated with each function/pathway versus the total number of genes (available in IPKB) involved in that particular function/pathway. The ratios may be affected by the variations in the total number of input identifiers. In order to find the significance of enrichment in a particular function, IPA calculates the significance value based on the measure of involvement of the gene in the input data set to their respective molecular functions/signaling pathways. Using the right-tailed Fisher's Exact Test, the p-value (significance) is calculated by comparing the number of user-specified genes of interest that participate in a given function or pathway, relative to the total number of occurrences of these genes in all functional/pathway annotations in the IPKB.

### Real-time PCR

To validate the microarray results, we carried out the real-time PCR for 25 genes and *beta-actin* gene was used as a reference. The primers used for amplifying each gene were listed in Table S18. cDNA was generated using the same purified RNA preparations from 72-hpf embryos (one biological repeat of wt and two biological repeats of *mib^ta52b^*) used in microarray, and two other biological repeats of reference RNA from 72 hpf and one biological repeat sample RNA from *mib^ta52b^* mutants. RT-PCR was carried out using the LightCycler® FastStart DNA Master^PLUS^ SYBR Green kit (Roche) and the Light Cycler machine as per the instructions of the manufacturer. The products of the RT-PCR were analyzed on the agarose gel electrophoresis for a single band of expected size. Relative cDNA amounts were calculated using the comparative C_T_ method as explained in the real-time PCR manual of Applied Biosystems and normalized to the expression of *beta-actin*.

### Analysis of correlation between microarray data and real-time PCR results

Subsequent to microarray and real-time PCR data analysis, an evaluation of linear correlation was performed for a set of 25 genes (Table 5), and the statistical significance of the correlation was determined using One-way ANOVA in SPS software. For the correlation analysis, the data input of the microarray was the fold change of expression and the data input of the real-time PCR was the ratio of relative expression for each gene. Both sets of ratios were obtained from the 72-hpf time point for the *mib^ta52b^* allele.

## Supporting Information

Table S1 List of significantly expressed genes (q = 0.0) in 72-hpf ta52b mutants generated with SAM.
(1.75 MB XLS)

Table S2 List of significantly expressed genes (q = 0.0) in 72-hpf m132 mutants generated with SAM.
(3.12 MB XLS)

Table S3 List of significantly expressed genes (q = 0.0) in 72-hpf tfi91 mutants generated with SAM.
(3.92 MB XLS)

Table S4 List of significantly expressed genes (q = 0.0, score(d)>4.0) common to all three mib mutant alleles at 72 hpf.
(0.09 MB XLS)

Table S5 List of significantly expressed genes (q = 0.0, score(d)>4.0) specific to ta52b mutants at 72 hpf.
(0.09 MB XLS)

Table S6 List of significantly expressed genes (q = 0.0, score(d)>4.0) specific to m132 mutants at 72 hpf.
(0.52 MB XLS)

Table S7 List of significantly expressed genes (q = 0.0, score(d)>4.0) specific to tfi91 mutants at 72 hpf.
(0.93 MB XLS)

Table S8 List of significantly expressed genes (q = 0.0) common to all three mib mutant alleles at 48 hpf.
(0.04 MB XLS)

Table S9 List of significantly expressed genes (q = 0.0) specific to ta52b mutants at 48 hpf.
(0.08 MB XLS)

Table S10 List of significantly expressed genes (q = 0.0) specific to m132 mutants at 48 hpf.
(0.09 MB XLS)

Table S11 List of significantly expressed genes (q = 0.0) specific to tfi91 mutants at 48 hpf.
(0.38 MB XLS)

Table S12 List of significantly expressed genes (q = 0.0) common at all three time points for ta52b mutants.
(0.05 MB XLS)

Table S13 List of significantly expressed genes (q = 0.0) specific to 24 hpf time point in ta52b mutants.
(1.01 MB XLS)

Table S14 List of significantly expressed genes (q = 0.0) specific to 48 hpf time point in ta52b mutants.
(0.03 MB XLS)

Table S15 List of significantly expressed genes (q = 0.0) specific to 72 hpf time point in ta52b mutants.
(1.07 MB XLS)

Table S16 List of significantly expressed genes (q = 0.0) common in at least two data sets.
(0.02 MB XLS)

Table S17 Zebrafish pancreas development related genes.
(0.02 MB XLS)

Table S18 Sequence of primers used in real-time PCR validation on microarray data.
(0.07 MB DOC)

Table S19 Genes predicted to be related to zebrafish pancreas development.
(0.02 MB XLS)

Table S20 Summary of biological and technical repeats.
(0.01 MB XLS)

Figure S1 Changes of expression levels in the canonical pathways. (A) The Notch signaling pathway and (B) the Wnt/β-catenin signaling pathway. The nodes in these pathways are highlighted with expression data from the *m132* data (red: up-regulated; green: down-regulated). Nodes with a histogram chart next to it represents gene expression in the i) *ta52b*, ii) *m132* and iii) *tfi91* data set from left to right. (TIF)
(1.71 MB TIF)
