# Supplementary material for: Genomewide Expression Analysis in Zebrafish mind bomb Alleles with Pancreas Defects of Different Severity Identifies Putative Notch Responsive Genes
Source: PLoS One. 2008 Jan 23;3(1):e1479. doi: 10.1371/journal.pone.0001479 (PMC2195453; doi:10.1371/journal.pone.0001479)
Supplement: Table S18 — Sequence of primers used in real-time PCR validation on microarray data. (0.07 MB DOC) [file pone.0001479.s018.doc]

**Table S18.** Sequence of primers used in real-time PCR validation on microarray data.

| **Genbank ID** | **Gene symbol** | **Primer designation** | **primer sequence (5’ – 3’)** | **GC content (%)** |
| --- | --- | --- | --- | --- |
| AF057040 | beta-actin1 | AF057040_F1 | tggatcagcaagcaggagtacg | 54.55 |
|  |  | AF057040_R1 | aggagggcaaagtggtaaacgc | 54.55 |
| BI891976 | zgc:56374 | BI891976_F1 | ggaccttcatgagcatggtgtc | 54.55 |
|  |  | BI891976_R1 | ctcccattccagacactgcatc | 54.55 |
| AF017266 | gad1 | gad1_F1 | gacacctgtgactccgtaagtg | 54.55 |
|  |  | gad1_R1 | gtcgcacgacatctggttcttg | 54.55 |
| AJ297822 | try | trypsin_F1 | gaccgtctctctgccttcaagc | 59.09 |
|  |  | trypsin_R1 | ttcctctgggcacagccataac | 54.55 |
| D21135 | isl1 | isl1_F1 | cggcgagacctttacaaatggc | 54.55 |
|  |  | isl1_R1 | tgggttgctgctgctggagttg | 59.09 |
| X97332 | her4 | her4_F1 | cagcagcatcgagaagctcaag | 54.55 |
|  |  | her4_R1 | gcgtgtgtttcagtggtctgag | 54.55 |
| X69088 | notch1a | notch-F1 | agacctgcgcttgaactcgatg | 54.55 |
|  |  | notch-R1 | gactccagcagacgtttagcag | 54.55 |
| U57975 | notch3 | notch3_F1 | acgctggctatgtcaccaatcc | 54.55 |
|  |  | notch3_R1 | gccagtcaaacggcacgcttac | 59.09 |
| AI522447 | dla | deltaA_F1 | ggacgggtttacaggcatgaac | 54.55 |
|  |  | deltaA_R1 | ggaacgtcgctggcaatttggg | 59.09 |
| AF109373 | opn1sw1 | opn1sw1_F1 | caggcagcctctcaactacatc | 54.55 |
|  |  | opn1sw1_R1 | gcacaggcggtaccaatgatcc | 59.09 |
| AW281193 |  | AW281193_F1 | ccagccatgcccatcccatcag | 63.64 |
|  |  | AW281193_R1 | cttgccgtgcttcatgtgcttg | 54.55 |
| BI709417 | prx-IV | prx-IV_F1 | ggttgtgtgtgacggtgcaaac | 54.55 |
|  |  | prx-IV_R1 | tcgtgcacacgatcactgaagg | 54.55 |
| BI887742 | sfrs5 | sfrs5_F1 | agccctcatgctcgtgaaagag | 54.55 |
|  |  | sfrs5_R1 | tgtgcgcactggtggtccatac | 59.09 |
| AF198033 | igfbp2 | AF198033_F1 | ctgtccgagtgcgcttcttacc | 59.09 |
|  |  | AF198033_R1 | gagcatctcggtgtgtaaacgc | 54.55 |
| AW019843 | sst1 | sst1_F1 | cacgcgtccgagcaaagagaac | 59.09 |
|  |  | sst1_R1 | gacagatcctctggctccagcg | 63.64 |
| AW282071 |  | AW282071_F1 | gaggctcagatcgacaaatgcc | 54.55 |
|  |  | AW282071_R1 | ggtagcggctggattggagatg | 59.09 |
| AB062116 | hsp70 | hsp70-F1 | cacggtgtttgatgccaagagg | 54.55 |
|  |  | hsp70-R1 | gcgacgactccagcatctttag | 54.55 |
| BI890045 | perp | perp-F1 | gctgcccttcatcggactactc | 59.09 |
|  |  | perp-R1 | tggagtggtgtgtgagactgtc | 54.55 |
| BM183964 | glo1 | glo1_F1 | acggcaactcagacccaagag | 57.14 |
|  |  | glo1_R1 | cagagagcaaccacccttcct | 57.14 |
| AF036326 | ins | insulin_F1 | cagcacctgtgtggatctcatc | 54.55 |
|  |  | insulin_R1 | aaggcctgtgtgcaaacaggtg | 54.55 |
| BI846588 |  | pappalysin2_F1 | gaggacggaagctggcagtttg | 59.09 |
|  |  | pappalysin2_R1 | gcagctgagatggatgtttggg | 54.55 |
| AF036325 | ipf1 | ipf1_F1 | caggcccttatatggtcgaggc | 59.09 |
|  |  | ipf1_R1 | ttgccgtgccgacgcaagattc | 59.09 |
| D38454 | zfIsl-3 | zfIsl-3_F1 | aggagcagctggtggagatgac | 59.09 |
|  |  | zfIsl-3_R1 | ccagctgttgaaaggcaggttg | 54.55 |
| AB006087 | spon1b | spon1b_F1 | gcggagagacattcgagatctg | 54.55 |
|  |  | spon1b_R1 | ggctggaggacagttggtcatg | 59.09 |
| BI533195 | igf2 | igf2_F1 | ggtggacgctctacagtttgtg | 54.55 |
|  |  | igf2_R1 | ctctgagcagcctttctttgcc | 54.55 |
| AY583322 | elastase A | ela_F1 | ggtggtgctcttcctgacaaac | 54.55 |
|  |  | ela_R1 | gaccttgtcaacccagtcagtg | 54.55 |

The nucleotide sequences are shown in a 5’ to 3’ orientation. Primers were designed using the primer design program in the website, <http://dbb.nhri.org.tw/primer/index.html>. The Genbank ID and Gene symbol were obtained using Zebrafish Chip Annotation Database (Unigene Build 85), <http://giscompute.gis.a-star.edu.sg/~govind/zebrafish/version2/>.
