# Supplementary figures and images for: Genomewide Expression Analysis in Zebrafish mind bomb Alleles with Pancreas Defects of Different Severity Identifies Putative Notch Responsive Genes
Source: PLoS One. 2008 Jan 23;3(1):e1479. doi: 10.1371/journal.pone.0001479 (PMC2195453; doi:10.1371/journal.pone.0001479)

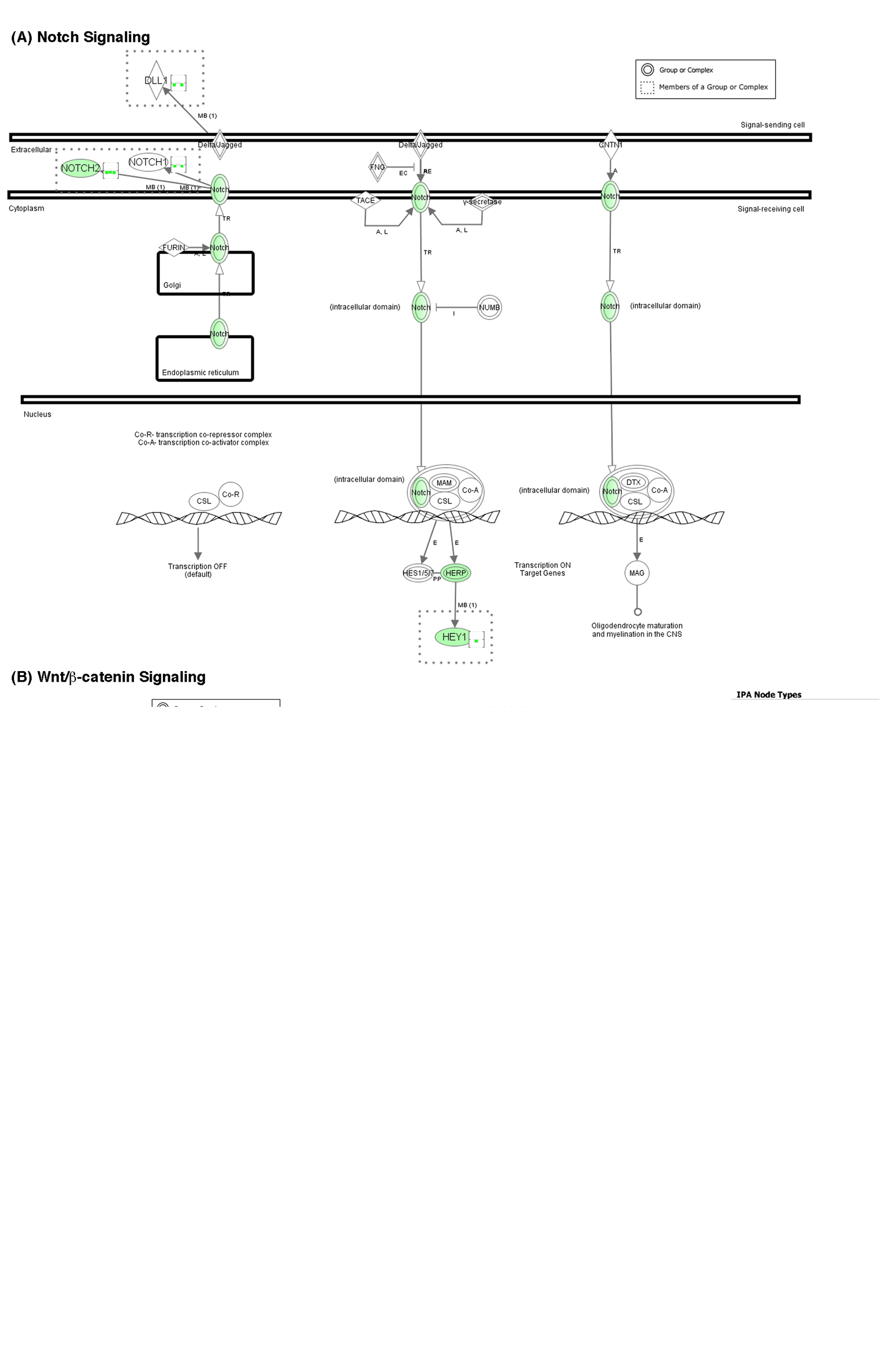

Supplement: Figure S1 — Changes of expression levels in the canonical pathways. (A) The Notch signaling pathway and (B) the Wnt/β-catenin signaling pathway. The nodes in these pathways are highlighted with expression data from the m132 data (red: up-regulated; green: down-regulated). Nodes with a histogram chart next to it represents gene expression in the i) ta52b, ii) m132 and iii) tfi91 data set from left to right. (TIF) (1.71 MB TIF) [file pone.0001479.s021.tif]
